# Utilization of a stabilized hyaluronic acid spacer in SBRT for retroperitoneal cancers: A case series and dosimetric analysis

**DOI:** 10.1016/j.ctro.2025.100943

**Published:** 2025-03-08

**Authors:** Shing Fung Lee, Nathanial Harris, Pui Lam Yip, Jenna Dean, Brayden Geary, George Koufogiannis, Melanie Bauer, Daryl Lim Joon, Farshad Foroudi, Ee Siang Choong, Michael Chao

**Affiliations:** a Department of Radiation Oncology, National University Cancer Institute, National University Hospital, Singapore; b Department of Medicine, Yong Loo Lin School of Medicine, National University of Singapore, Singapore; c Olivia Newton John Cancer Wellness and Research Centre, Austin Hospital, Heidelberg, Victoria, Australia; d Ringwood Private Hospital, Melbourne, Victoria, Australia; e Monash University, Melbourne, Victoria, Australia; f Genesis Care Victoria, Ringwood Private Hospital, Ringwood East, Victoria, Australia; g University of Melbourne, Melbourne, Victoria, Australia

**Keywords:** Stereotactic body radiation therapy, Hyaluronic acid, Retroperitoneal neoplasms, Renal cell carcinoma, Organ at risk

## Abstract

**Objectives:**

To evaluate the clinical indications, post-operative complications, and dosimetric impact of stabilized hyaluronic acid (sHA) spacer insertion in the retroperitoneum for stereotactic body radiation therapy (SBRT) of retroperitoneal cancers.

**Patients and methods:**

This retrospective study analyzed two cases of SBRT for retroperitoneal lesions: a primary left renal cell carcinoma and an oligometastatic renal cell carcinoma (RCC) lesion in the right adrenal gland. In both cases, a sHA spacer was percutaneously inserted between the bowel and the lesion. The spacer was placed under laparoscopic and ultrasound guidance for the renal tumor, and under CT guidance for the adrenal lesion. The prescribed dose was 42 Gy in three fractions for the primary renal lesion and 40 Gy in five fractions for the adrenal lesion. Dosimetric parameters were evaluated in two planning scenarios: (1) without the spacer, adhering to OAR constraints, and (2) with the spacer.

**Results:**

Spacer insertion for the primary left renal cancer significantly improved PTV D95% from 67.9 % to 99.5 % of the prescribed dose, a 46.5 % increase. PTV D99% increased from 54.5 % to 86.7 %, reflecting a 59.1 % improvement. The large bowel’s maximum dose was reduced from 28.2 Gy to 24.4 Gy, and the volume receiving 24 Gy was minimal with the spacer (0.06 cc). For the right oligometastatic adrenal lesion, despite a fourfold increase in tumor volume, post-spacer dosimetry showed improved PTV coverage without exceeding OAR constraints. No post-operative complications were observed in either case.

**Conclusion:**

This is the first report on sHA spacer use in the retroperitoneum for SBRT. Spacer insertion near the large bowel significantly improves dosimetry, enabling higher doses to targets while keeping OAR doses within safe limits. Further research with a larger patient population is required to assess safety and for optimization of the technique for spacer placement in order to enhance clinical outcomes.

## Highlights

• Use of stabilized hyaluronic acid (sHA) spacers in SBRT for retroperitoneal cancers.
• Spacer application improved PTV coverage while keeping OAR doses within safe limits.
• Feasibility and dosimetric benefits in kidney and adrenal lesions.
• Laparoscopic and CT-guided spacer placement procedures were safe and well-tolerated.
• Spacer use offers potential for applications in high-dose RT for complex anatomies.

## Introduction

Renal cell carcinoma (RCC) is traditionally considered to be a radioresistant tumor based on preclinical studies and negative clinical trials using conventionally fractionated radiation therapy (RT).[1] SBRT enables delivery of highly precise ablative doses to targets while sparing adjacent healthy tissues.[2] However, in the abdominal and retroperitoneal regions, achieving adequate planning target volume (PTV) coverage without exceeding organ at risk (OAR) constraints is challenging due to the proximity of luminal structures.

Stabilized hyaluronic acid (sHA) spacers are increasingly used in prostate cancer treatment to create physical separation between the prostate and rectum, reducing rectal radiation exposure.[3] However, complications of gel spacer insertion, such as pain, abscess formation, and rare cases of rectal wall infiltration, highlight the importance of proper placement and procedural expertise.[[4], [5], [6]] Unlike the relatively stable anatomy of the prostate-rectum interface, retroperitoneal applications pose additional challenges due to the dynamic position of abdominal organs. While these biocompatible and biodegradable spacers have potential applications in other anatomical sites, their use in SBRT for retroperitoneal cancers remains underexplored.[7].

We report two cases of retroperitoneal cancers where sHA spacers were used to separate target lesions from the adjacent large bowel, facilitating higher radiation doses while maintaining OAR safety. A dosimetric analysis was performed pre- and post-spacer insertion, evaluating scenarios with 0, 3, and 5 mm PTV/ planning organ at risk volume (PRV) margins. This study highlights the potential of sHA spacers to enhance therapeutic outcomes for retroperitoneal cancers.

The dosimetric analysis details are presented in Appendix 1, with Tables S1 and S2 outlining the planning goals and OAR dose constraints for renal and adrenal cases, respectively.

## Case 1: Left renal lesion

An 84-year-old male diagnosed with primary left renal cancer in December 2023. The tumor, located at the mid-to-lower pole, measured 3.8 × 3.4 cm on diagnostic computed tomography (CT), with no evidence of metastasis. Due to comorbidities, he was deemed medically inoperable and referred for SBRT.

**Fig. 1:**
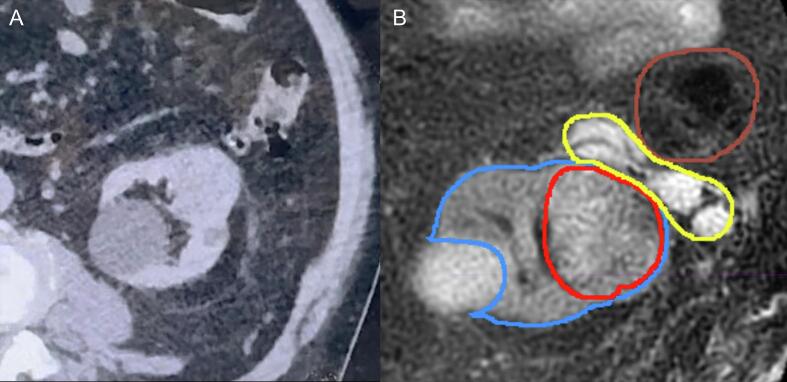
**Pre- and post-spacer insertion CT scans for the primary left renal tumor.** (A): Pre-spacer insertion CT showing the left renal tumor closely adjacent to the large bowel, with minimal separation. (B): Post-spacer insertion scan with delineation of key structures. The blue outline represents the left kidney. The left renal tumor (outlined in red) is now separated from the large bowel (outlined in brown) by the sHA spacer (outlined in yellow). The spacer creates a safe distance between the large bowel and the high-dose radiation field.

To address the tumor’s close proximity to the large bowel, a sHA spacer was laparoscopically implanted under general anesthesia in January 2024 (Fig. 1). The procedure was completed without complications, and the patient was discharged the following day.

**Fig. 2:**
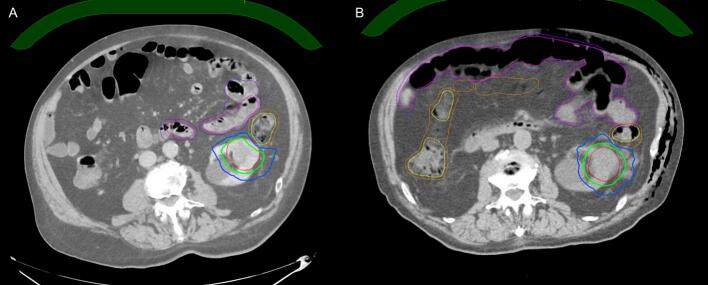
**Comparative CT images showing the planning target volume (PTV) with a 5 mm margin and planning organ at risk volume (PRV) with a 3 mm margin for the primary left renal tumor before and after spacer and non-spacer insertion. (A) Non-spacer plan:** CT simulation image showing the contours of the gross tumor volume (GTV, red), PTV (cyan), large bowel (yellow), and large bowel PRV (orange). The prescribed dose (42 Gy) and 28.2 Gy isodose lines are represented by the thick green and blue line respectively. **(B) Spacer plan:** CT simulation image demonstrating the use of a sHA spacer to create separation between the GTV (red), PTV (cyan), large bowel (yellow), and large bowel PRV (orange). The prescribed dose (42 Gy) and 28.2 Gy isodose lines are represented by the thick green and blue line respectively.

SBRT was delivered using the Elekta Unity magnetic resonance (MR) linac, prescribed at 42 Gy in three fractions. Treatment planning incorporated CT and magnetic resonance imaging (MRI) simulations, with motion assessment via cine MRI during the MRI simulation and confirmed with motion assessment on the 4-dimensional CT. Additional history and planning details are provided in Appendix 2. Spacer insertion enabled improved dosimetry: PTV D95% increased from 67.9 % to 99.5 % (46.5 % improvement), and PTV D99% increased from 54.5 % to 86.7 % (59.1 % improvement) (Table 1, Fig. 2). Large bowel D0.035 cc was reduced from 28.2 Gy to 24.4 Gy, and V24Gy was minimized to 0.06 cc. With larger PRV margins, spacer benefits were more pronounced; at 5 mm PRV, PTV D95% improved from 24.8 Gy to 39.4 Gy (58.8 % increase) (Table S3).

**Table 1:** Dosimetric parameters of the originally delivered plans.

**Dosimetric parameters**	**Optimal constraint/goal**	**Acceptable constraint**	**Without spacer**	With spacer*	**Relative difference (%)**	**Absolute difference**
**Left kidney primary tumor (prescription 42 Gy in 3 Fr)**						
GTV, cc	−		41.2	48.5	17.7 %	7.3
†PTV, cc	−		81.9	94.4	15.3 %	12.5
PTV mean, Gy	−		46.7	50.5	8.1 %	3.8
PTV D95%Gy (%)	>42 Gy (i.e. 100 %)	−	28.5 (67.9)	41.8 (99.5)	46.5 %	13.3 (31.6)
PTV D99%Gy (%)	>42 Gy (i.e. 100 %)	>39.9 Gy (i.e. 95 %)	22.9 (54.5)	36.4 (86.7)	59.1 %	13.5 (32.2)
Large Bowel D0.035ccGy	<28.2 Gy	−	28.2	24.4	−13.5 %	−3.8
Large Bowel V24Gy cc	<20 cc	−	0.2	0.06	−70.0 %	−0.14
‡Large Bowel PRV D0.035 cc, Gy	<28.2 Gy	−	28.1	27.9	−0.7 %	−0.2
**Right adrenal metastasis (prescription 40 Gy in 5 Fr)**						
GTV, cc	−		5	19	280.0 %	14
†PTV, cc	−		28.5	53	86.0 %	24.5
PTV mean, Gy	−		39.7	39.4	−0.8 %	−0.3
PTV D95%, Gy (%)	>40 Gy (i.e. 100 %)	−	28.3 (70.8)	30.6 (76.5)	8.1 %	2.3 (5.7)
PTV D99%, Gy (%)	>38 Gy (i.e. 95 %)	−	25.7 (67.6)	27.6 (72.6)	7.4 %	1.9 (5.0)
Large Bowel D0.035 cc, Gy	≤32 Gy	≤34 Gy	33.6	33.9	0.9 %	0.3
Large Bowel D20cc, Gy	<25 Gy	−	18.8	20.6	9.6 %	1.8
‡Large Bowel PRV D0.035 cc, Gy	<38 Gy	−	37.4	37.1	−0.8 %	−0.3

Abbreviations: GTV, gross tumor volume; Gy, Gray; PRV, planning organ at risk volume; PTV, planning target volume.

* The right adrenal lesion quadrupled in volume over 3 months as noted in the pre- and post-spacer CT simulation scan.

† PTV margin is 5 mm.

‡ PRV margin is 3 mm.

The patient tolerated SBRT well, with only grade 1 fatigue that resolved within a few days. At the seven-months, renal function was stable, and imaging showed no disease progression.

## Case 2: Right adrenal lesion

A 72-year-old male with metastatic RCC was noted in November 2020 to have a right adrenal gland lesion that had increased in size from 8 mm to 18 mm over the past seven months. SBRT was planned; however, the lesion’s proximity to the large bowel posed a challenge. To create separation, an sHA spacer was implanted under CT guidance without anesthesia (Fig. S1). The procedure was completed without complications.

**Fig. 3:**
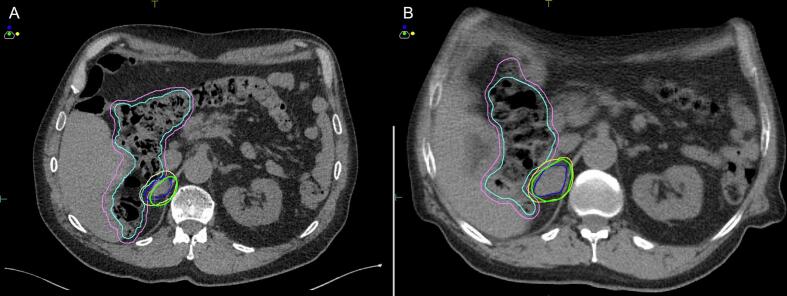
**Comparative CT images showing the planning target volume (PTV) with a 5 mm margin and planning organ at risk volume (PRV) with a 3 mm margin for the right adrenal lesion before and after spacer and non-spacer insertion. (A) Non-spacer plan:** CT simulation image showing the contours of the gross tumor volume (GTV, blue), PTV (yellow), large bowel (cyan), and large bowel PRV (light purple). The prescribed dose (40 Gy) isodose is indicated by the thick green line. **(B) Spacer plan:** CT simulation image demonstrating the use of a sHA spacer to create separation between the GTV (blue), PTV (yellow), large bowel (cyan), and large bowel PRV (light purple). The prescribed dose (40 Gy) isodose is indicated by the thick green line.

The tumor quadrupled in tumor volume over three months as noted in the pre- and post-spacer insertion CT simulation scans. Due to this increase in size, full PTV coverage was not achievable in either plan. However, the spacer facilitated improved relative coverage while keeping OAR doses within tolerance. The prescribed dose of 40 Gy in five daily fractions was delivered within these constraints (Fig. S2, Table 1). The lesion was visualized on both CT and MRI, which facilitated accurate treatment planning (Figs. S3 and Fig. 3). Additional history and planning details are provided in Appendix 3. SBRT was delivered using a CT linear accelerator with daily cone beam CT (CBCT), utilizing 6 megavoltage Flattening Filter-Free volumetric modulated arc therapy (VMAT) with two 360-degree arcs. This approach enabled faster treatment delivery but provided less optimal soft tissue resolution compared to MRI-guided systems. The treatment was completed in May 2021.

Dosimetry analysis showed significant improvements in PTV coverage post-spacer (Table 1). As the large bowel PRV margin increased, the benefit of the spacer became more pronounced. PTV V40Gy increased from 62.2 % to 73.1 % with no PRV (a 17.6 % improvement) and from 44.6 % to 66.2 % with a 5 mm PRV, reflecting a 48.5 % improvement. All other OAR doses, including those to the duodenum, spinal cord, and liver, remained within tolerance (Table S4).

The patient experienced grade 1 diarrhea, which resolved within two weeks. At the November 2023 follow-up, the disease was stable without adrenal insufficiency or hypertension.

## Discussion

This retrospective study reports on two cases where a sHA spacer was used to separate the large bowel from a primary RCC lesion and a metastatic RCC adrenal lesion, respectively. Comparative dosimetric analyses were performed to evaluate the differences in radiation dose distribution before and after spacer insertion by replanning with varying PRV margins for the large bowel and PTV margins. This approach accounts for practical considerations related to internal organ movement and setup uncertainty, demonstrating the dosimetric advantage of using spacers when critical organs are close to the target.

The widespread adoption of gel spacers in prostate cancer is largely due to their ease of implantation and favorable safety profiles, particularly when compared to other spacer materials such as balloons or catheters.[[8], [9], [10], [11], [12]] Notably, gel spacers can be compositionally distinct. The sHA spacer used in our study differs from polyethylene glycol-based hydrogels, such as SpaceOAR, and may therefore have a different safety profile.[[4], [5], [6]] Additionally, spacer stability is typically maintained throughout the treatment course.[13] However, complications such as rectal wall infiltration and spacer migration emphasize the need for precise placement to ensure stability during treatment.[[4], [5], [6]] These challenges are magnified in retroperitoneal applications, where organ mobility and complex anatomy may pose additional risks.[8,11] Spacer misplacement or migration in retroperitoneal SBRT could delay treatment and compromise clinical outcomes. Despite these challenges, the results of this study demonstrate the feasibility and dosimetric advantages of sHA spacers in retroperitoneal SBRT, providing consistent target separation and sparing critical structures like the large bowel. Future research should refine insertion techniques, patient selection, and planning workflow.

The MR-linac offered superior soft tissue visualization and enabled precise online adaptive planning, particularly for the left renal case.[14] However, the absence of gating in this system posed challenges in managing respiratory motion, requiring careful consideration of PTV margins to ensure adequate coverage. The treatment time on the MR-linac was longer due to the online adaptive workflow and 13-field step-and-shoot IMRT delivery, which may add to patient discomfort. In contrast, the conventional linac for the adrenal case, utilizing dual arc VMAT, allowed for faster treatment delivery. However, the reliance on CBCT for localization offered less optimal soft tissue resolution compared to MRI.[14].

Our experience with the use of a sHA spacer in SBRT for treating renal and adrenal lesions demonstrated that this approach offers a crucial advantage by providing physical separation between the tumor and the large bowel. This separation is particularly important because high doses of radiation delivered to the bowel during SBRT can lead to rare but serious complications. One such complication is the formation of a nephrocolic fistula, a pathological connection between the kidney and the colon, which can result in significant morbidity and mortality.[15] The spacer buffers the bowel from high dose radiation, reducing adverse risk. Daily verification imaging in both cases showed that the sHA in the retroperitoneum remained constant in shape and position throughout the SBRT courses, providing consistent physical separation. By ensuring that the radiation dose is concentrated on the tumor while sparing adjacent organs, the spacer not only enhances the safety of SBRT but also maintains its therapeutic efficacy.

Despite the significant increase in the right adrenal PTV due to tumor growth, we successfully delivered the prescribed radiation dose to the target area while protecting surrounding OARs. This was particularly significant for the large bowel, which remained within safe dose constraints post-sHA spacer insertion. Besides, the sHA spacer's MRI visibility facilitated re-planning by enabling accurate contouring of target volumes and OARs.

## Conclusion

To the best of our knowledge, this is the first report demonstrating the feasibility of using a sHA spacer for retroperitoneal SBRT, highlighting a novel application of an established technology. Spacer placement successfully created a separation between retroperitoneal tumors and adjacent critical structures, significantly improving PTV dosimetry while maintaining OAR doses within constraints. This approach enabled the safe delivery of high-dose SBRT in anatomically complex regions. Our findings suggest that this technique may have broader applications beyond renal and adrenal cases, potentially benefiting patients with retroperitoneal sarcomas and pancreatic tumors, where high or ablative doses of RT may be indicated. Further research is warranted to spacer placement techniques, assess safety profiles, and evaluate its clinical impact in larger patient cohorts.

## Declaration of competing interest

The authors declare the following financial interests/personal relationships which may be considered as potential competing interests: MC reported receiving consultant fees from Teleflex, unrelated to the submitted work. No other conflicts of interest were declared.

## Data Availability

Research data are not available at this time due to patient privacy.
