# Supplementary material for: Utilization of a stabilized hyaluronic acid spacer in SBRT for retroperitoneal cancers: A case series and dosimetric analysis
Source: Clin Transl Radiat Oncol. 2025 Mar 8;52:100943. doi: 10.1016/j.ctro.2025.100943 (PMC11950742; doi:10.1016/j.ctro.2025.100943)
Supplement: Supplementary Data 1 [file mmc1.docx]

**Appendix 1.** Dosimetric Analysis.

This retrospective study analyzed two cases of SBRT for retroperitoneal lesions: one involving a primary left RCC, and the other a right adrenal oligometastatic lesion, from a primary RCC. Both patients were selected due to their lesions’ proximity to the critical OARs, specifically the large bowel, which necessitated the use of a sHA spacer to improve dosimetric outcomes. The spacer insertion procedure, RT planning, and delivery were detailed for both cases. Additionally, the safety and tolerability of the spacer insertion and SBRT were evaluated for each patient.

Comparative dosimetric analyses were performed to evaluate radiation dose distribution before and after spacer insertion. Replanning scenarios were conducted with varying PRV margins (0, 3, and 5 mm) for the large bowel and PTV margins (0, 3, and 5 mm). The impact of the sHA spacer on dose coverage to the PTV and doses to OARs were assessed. Key dosimetric parameters included PTV D95%, PTV D99%, and the maximum doses to the large bowel due to its critical proximity to the target lesions. The analysis also considered other OARs (with or without PRV) such as the duodenum, spinal cord, and liver. PRV margins are routinely applied in CT-based linac SBRT at our institution to account for inter-fraction organ motion. While predefined PRV expansion is not required in the MR-linac setting due to daily adaptive planning, it was included in this study to standardize dosimetric comparisons and illustrate the spacer's impact. This approach enabled a comprehensive assessment of how the spacer and PRV margins influence dose distribution and target coverage across different planning scenarios.

The dosimetric parameters, goal, and constraints for the two cases are shown in Tables S1 and S2, respectively. The primary outcomes of this study were improvements in PTV coverage and reductions in doses to adjacent OARs.

This study was conducted in compliance with institutional guidelines and ethical standards. The patients provided written informed consent for the publication of their clinical details and accompanying images.
