# Supplementary material for: Utilization of a stabilized hyaluronic acid spacer in SBRT for retroperitoneal cancers: A case series and dosimetric analysis
Source: Clin Transl Radiat Oncol. 2025 Mar 8;52:100943. doi: 10.1016/j.ctro.2025.100943 (PMC11950742; doi:10.1016/j.ctro.2025.100943)
Supplement: Supplementary Data 2 [file mmc2.docx]

**Appendix 2.** Radiation Therapy Simulation and Treatment Planning for the Left Renal Lesion (Case 1).

From diagnostic CT, we identified the challenge of the left kidney lesion being in close proximity to a large bowel loop, which could limit the dose delivered to the tumor. To mitigate this, a sHA spacer was implanted under direct visual guidance using laparoscopic and ultrasound imaging by a surgeon under general anesthesia in January 2024 (Figure 1). A total of 12ml was implanted. The procedure was completed without post-procedural complications, and the patient remained hospitalized overnight for observation.

SBRT was delivered using the Elekta Unity magnetic resonance (MR) linac. The treatment planning system used was Monaco TPS (Version 5.51.11; Elekta AB). For treatment planning, both CT and MRI simulations were performed. The MRI simulation included T2 3D navigated, T2 2D MultiVane, T2 2D MultiVane fat saturation, T1 3D Vane mDixon, diffusion-weighted imaging, and coronal and sagittal balanced fast field echo (bFFE) cine images to assess motion and verify position. These MRI images were fused with the CT images to aid in delineating the left renal lesion, OARs, and the distribution of the sHA spacer.

A 4DCT was acquired to evaluate tumor and organ motion, and the mean image was used correlating with the 3D Vane and T1 3D online planning images for treatment planning. No maximum intensity projection was generated, and an internal target volume was not explicitly created. Instead, motion assessment was performed on cine MRI during the MRI simulation and verified with 4DCT.

The simulation protocol involved using a T-shaped Elekta BodyFix vacuum mold, which supported the arms positioned above the head down to the mid-thighs, along with a Kneefix cushion abutting the vacuum bag for added stability. The patient was advised to fast for two hours before simulation to minimize potential changes in stomach position.

For daily localization, b3D Vane and T1 3D MRI sequences were used for online adaptation and verification prior to treatment. Additionally, bFFE real-time motion monitoring was performed to ensure the target remained within the prescribed margins throughout treatment. SBRT was delivered in free-breathing mode. The absence of gating required careful selection of PTV margins to account for respiratory motion. The daily online adaptive workflow allowed for real-time anatomical assessment, facilitating necessary adjustments to maintain PTV coverage and OAR constraints, particularly in response to daily changes in bowel position.

Abdominal compression, which is the standard practice for Unity MR-linac treatment in the abdomen, was not used, as the patient was unable to tolerate it due to discomfort following spacer insertion.
