# Supplementary material for: Utilization of a stabilized hyaluronic acid spacer in SBRT for retroperitoneal cancers: A case series and dosimetric analysis
Source: Clin Transl Radiat Oncol. 2025 Mar 8;52:100943. doi: 10.1016/j.ctro.2025.100943 (PMC11950742; doi:10.1016/j.ctro.2025.100943)
Supplement: Supplementary Data 3 [file mmc3.docx]

**Appendix 3.** Radiation Therapy Simulation and Treatment Planning for the Right Adrenal Lesion (Case 2).

In November 2020, imaging revealed that the right adrenal gland lesion had increased from 8 mm to 18 mm over a span of 7 months. SBRT was planned, but due to the lesion’s proximity to the large bowel, a sHA spacer was implanted under CT guidance by an interventional radiologist in the prone position without anesthesia (Figure S1). A total of 18ml was inserted, and the procedure was completed without any post-procedural complications or symptoms. The patient did not require hospitalization and was discharged the same day.

For treatment planning, both CT and MRI simulations were performed. The MRI included T1- and T2-weighted sequences, which were fused with the CT images to aid in target and OAR delineation and visualize the sHA spacer. The treatment planning system used was Monaco TPS (Version 6.1; Elekta AB) for the conventional linac.

The simulation protocol involved a Bodyfix vacuum bag covering the patient from the arms to the mid-thighs in the supine position, with arms positioned above the head and a Kneefix cushion for additional stability.

4D CT was acquired to assess tumor and organ motion. The average scan was generated in MIM and used as the planning CT. No maximum intensity projection was generated, and an internal target volume was not created. Motion evaluation from the 4D CT confirmed limited tumor displacement, and free breathing was deemed appropriate, as the patient was unable to tolerate end-expiration breath-hold.

For daily image-guided radiation therapy, the workflow consisted of an initial 4D CBCT, where all translational shifts were applied. A verification 4D CBCT was acquired to assess setup accuracy and ensure OAR doses remained within tolerance. If the 4D CBCT quality was insufficient to clearly visualize surrounding OARs, a static CBCT was used for verification. Intra-fraction imaging (IFI) was performed mid-treatment to confirm that the tumor remained in the correct position, ensuring that no more than 10 minutes elapsed between verification scans. A post-treatment IFI was also acquired to assess final positioning.

During the simulation, tumor progression was noted, with the lesion increasing in volume from 4.84 cc to 19.24 cc over a three-month interval between the initial and post-spacer CT simulation. This substantial growth posed an additional challenge to the planning process. However, the spacer created sufficient separation between the adrenal lesion and the large bowel, allowing the prescribed dose to be safely delivered.
