# Supplementary material for: Utilization of a stabilized hyaluronic acid spacer in SBRT for retroperitoneal cancers: A case series and dosimetric analysis
Source: Clin Transl Radiat Oncol. 2025 Mar 8;52:100943. doi: 10.1016/j.ctro.2025.100943 (PMC11950742; doi:10.1016/j.ctro.2025.100943)

**Figure S1.** **CT imaging of the patient’s bowel and adrenal metastatic lesion before (A), and after sHA spacer placement (B). The** CT images illustrate the positioning and effects of an 18 ml sHA spacer in a patient with a rapidly enlarging adrenal metastatic lesion. The image on the left (A) shows the anatomical structures before sHA spacer insertion, with the large bowel near the tumor. The image on the right (B) displays the anatomical changes after the sHA spacer placement, demonstrating the increased separation between the tumor and the large bowel. This strategic placement allowed for the delivery of a high radiation dose to the enlarged tumor while adhering to the dose constraints for the surrounding OARs, including the large bowel.


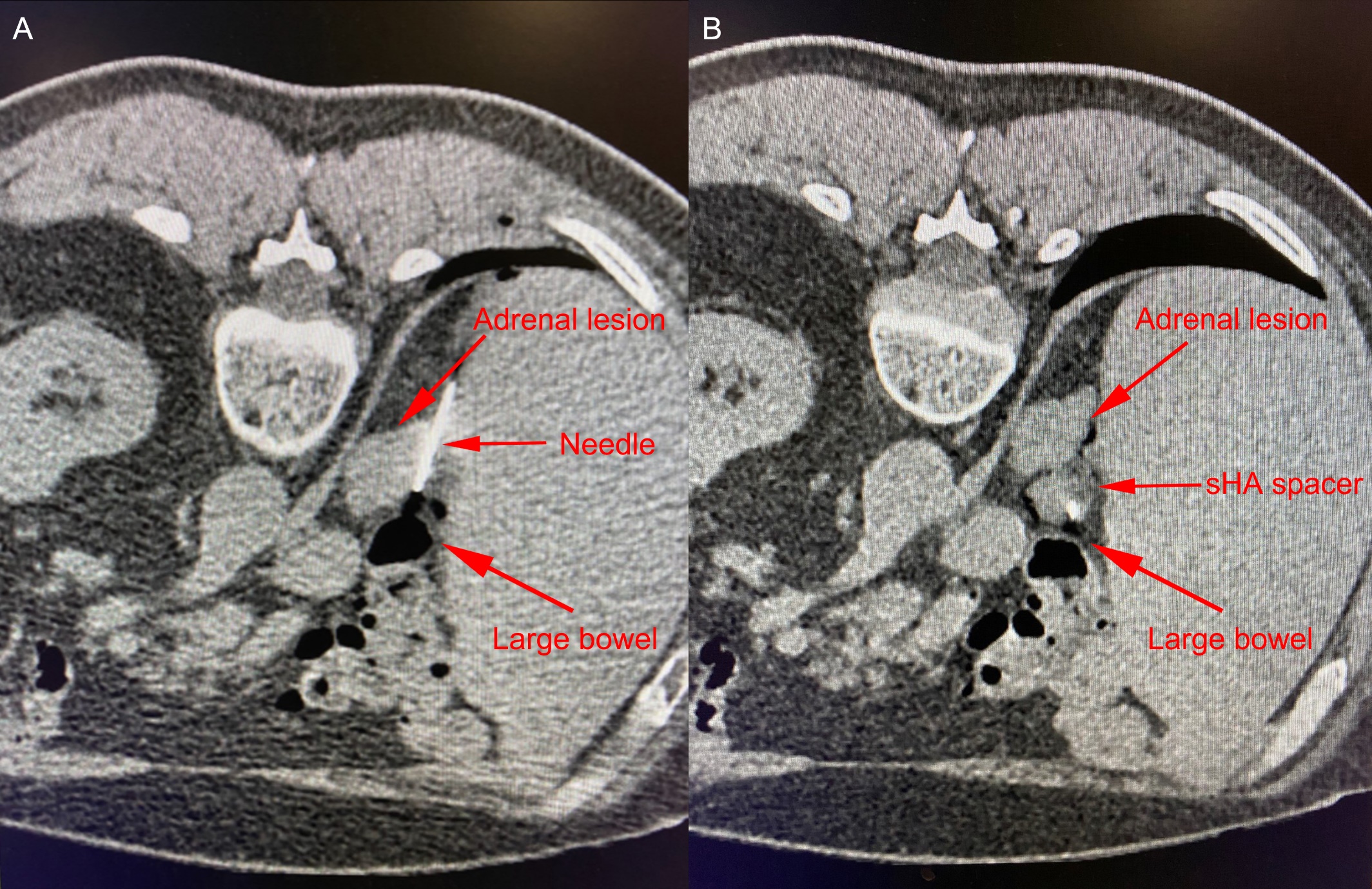

Supplement: Supplementary Data 4 [file mmc4.docx]
