# Supplementary material for: Utilization of a stabilized hyaluronic acid spacer in SBRT for retroperitoneal cancers: A case series and dosimetric analysis
Source: Clin Transl Radiat Oncol. 2025 Mar 8;52:100943. doi: 10.1016/j.ctro.2025.100943 (PMC11950742; doi:10.1016/j.ctro.2025.100943)
Supplement: Supplementary Data 5 [file mmc5.docx]

**Figure S2
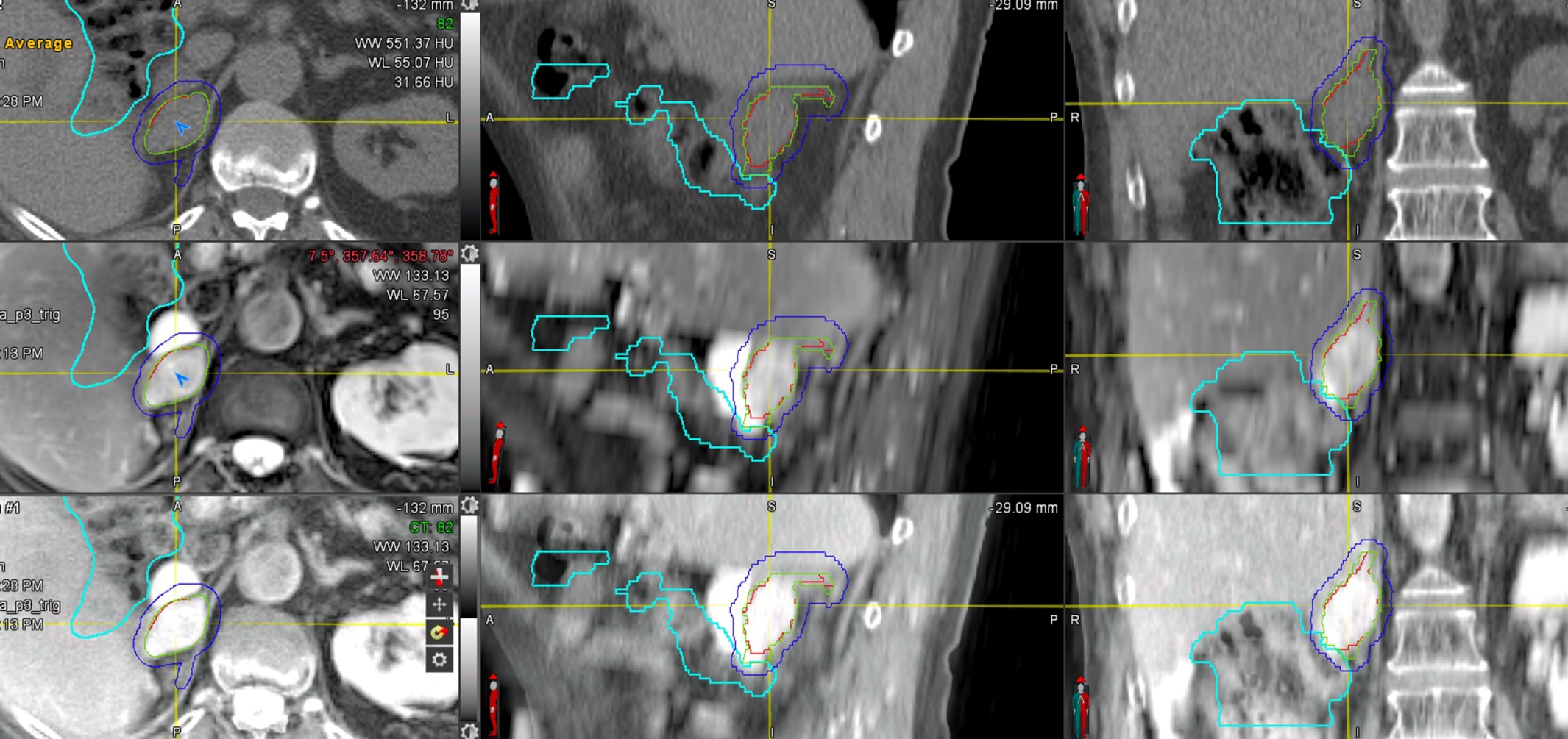
.** **Planning CT images for the right adrenal lesion post-spacer insertion.** Planning CT images for the right adrenal lesion after the insertion of the sHA spacer, with contourings displayed in axial, sagittal, and coronal views. The adrenal metastasis is outlined in red, the PTV in blue, and the large bowel in cyan. These images demonstrate that the spacer effectively distances the large bowel from the high-dose radiation field.
