# Supplementary figures and images for: Utilization of a stabilized hyaluronic acid spacer in SBRT for retroperitoneal cancers: A case series and dosimetric analysis
Source: Clin Transl Radiat Oncol. 2025 Mar 8;52:100943. doi: 10.1016/j.ctro.2025.100943 (PMC11950742; doi:10.1016/j.ctro.2025.100943)

**Figure S3
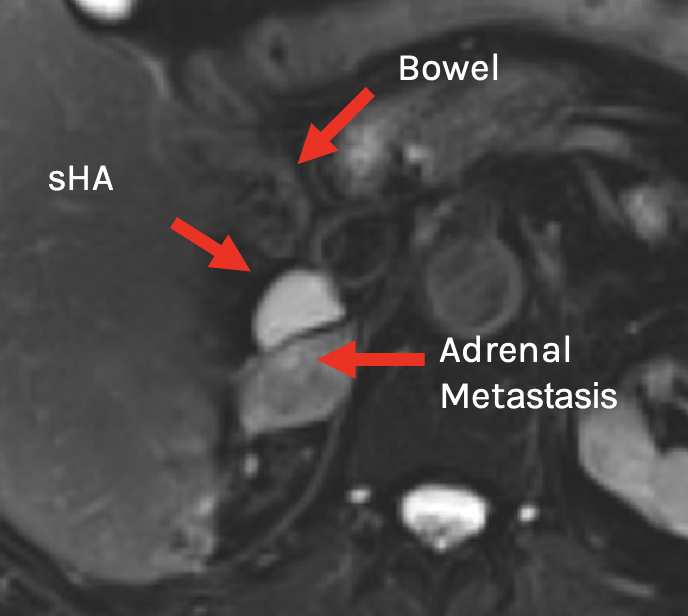
.** **MRI simulation showing right adrenal metastasis and spacer.**

Supplement: Supplementary Data 6 [file mmc6.docx]
