# Supplementary material for: Utilization of a stabilized hyaluronic acid spacer in SBRT for retroperitoneal cancers: A case series and dosimetric analysis
Source: Clin Transl Radiat Oncol. 2025 Mar 8;52:100943. doi: 10.1016/j.ctro.2025.100943 (PMC11950742; doi:10.1016/j.ctro.2025.100943)
Supplement: Supplementary Data 7 [file mmc7.docx]

**Table S2.** Overview of the dosimetric parameters, goal, and constraints for planning SBRT for the right adrenal metastasis.

| **Target/OAR** | **Parameter** | **Optimal constraint/goal** | **Acceptable constraint** |
| --- | --- | --- | --- |
| **GTV/ITV** | D99 | ≥38Gy | - |
| **PTV** | D95 | ≥40Gy | - |
|  | D99 | ≥38Gy | - |
|  | V40Gy | Recorded | - |
|  | D0.035cc | ≤55Gy | - |
| **Large bowel** | D0.035cc | ≤32Gy | ≤34Gy |
|  | D20cc | ≤25Gy | - |
| **Large bowel PRV** | D0.035cc | ≥38Gy | - |
| **Small bowel** | D0.035cc | ≤30Gy | ≤32Gy |
|  | D5cc | ≤19.5Gy | - |
| **Duodenum** | D0.035cc | ≤30Gy | - |
|  | D5cc | ≤18.5Gy | - |
|  | D10cc | ≤12.5Gy | - |
| **Duodenum PRV (5mm)** | D0.035cc | ≤30Gy | ≤32Gy |
| **Right chest wall** | D0.035cc | ≤50Gy | - |
|  | D70cc | ≤30Gy | - |
| **IVC** | D0.035cc | ≤47Gy | - |
| **Spinal canal** | D0.035cc | ≤25Gy | ≤28Gy |
| **Spinal canal PRV (2mm)** | D0.035cc | ≤25Gy | ≤28Gy |
| **Left kidney** | D0.035cc | ≤50Gy | - |
|  | D10% | ≤10Gy | - |
|  | Dmean | ≤10Gy | ≤12Gy |
| **Liver** | V10Gy | Recorded | - |
| **Liver minus GTV** | Dmean | ≤15Gy | ≤15.2Gy |
| **Skin rind (5mm subcutis)** | D0.035cc | ≤39.5Gy | - |
|  | D10cc | ≤36.5Gy | - |

Abbreviations: CTV, clinical target volume; GTV, gross tumor volume; ITV, internal target volume; IVC, inferior vena cava; PRV, planning risk volume; PTV, planning target volume; VxGy, volume receiving x Gy.
