# Supplementary material for: Utilization of a stabilized hyaluronic acid spacer in SBRT for retroperitoneal cancers: A case series and dosimetric analysis
Source: Clin Transl Radiat Oncol. 2025 Mar 8;52:100943. doi: 10.1016/j.ctro.2025.100943 (PMC11950742; doi:10.1016/j.ctro.2025.100943)
Supplement: Supplementary Data 8 [file mmc8.docx]

**Table S3.** Comparative dosimetric analysis for the left renal lesion using different PTV and large bowel PRV margins.

| **Plans with different PTV and PRV margins with and without spacer** | | | **Targets** | | | | | **Large bowel** | | **Large bowel PRV** | **Small bowel** | | **Small bowel PRV** | **Thecal sac** | **Thecal sac PRV** | **Skin rind** | | **Aorta** |
| --- | --- | --- | --- | --- | --- | --- | --- | --- | --- | --- | --- | --- | --- | --- | --- | --- | --- | --- |
|  |  |  | **GTV/ITV D99% (Gy)** | **PTV D95% (Gy)** | **PTV D99% (Gy)** | **PTV V42Gy (%)** | **D0.035cc (Gy)** | **D0.035cc (Gy)** | **V24Gy (cc)** | **D0.035cc (Gy)** | **D0.035cc (Gy)** | **V17.7Gy (cc)** | **D 0.035cc (Gy)** | **D0.035cc (Gy)** | **D0.035cc (Gy)** | **D0.035cc (Gy)** | **D10cc (Gy)** | **D0.035cc (Gy)** |
| No large bowel PRV | 0mm PTV expansion from GTV | No spacer | 42.58 | 45.73 | 42.58 | 99.30 | 58.12 | 28.05 | 0.87 | - | 23.03 | 2.72 | - | 7.85 | 8.30 | 23.9 | 9.79 | 9.49 |
|  |  | Spacer | 42.96 | 45.13 | 42.96 | 99.59 | 56.91 | 26.41 | 0.41 | - | 24.05 | 3.44 | - | 4.39 | 5.00 | 15.64 | 10.23 | 7.72 |
|  |  | Difference (%) | 0.9 | -1.3 | 0.9 | 0.3 | -2.1 | -5.8 | -52.9 | - | 4.4 | 26.5 | - | -44.1 | -39.8 | -34.6 | 4.5 | -18.7 |
|  | 3mm PTV expansion from GTV | No spacer | 43.30 | 40.83 | 32.57 | 93.40 | 58.11 | 27.95 | 0.86 | - | 22.96 | 5.92 | - | 7.88 | 8.26 | 21.67 | 9.88 | 9.39 |
|  |  | Spacer | 49.92 | 46.01 | 43.75 | 99.75 | 57.95 | 27.93 | 1.55 | - | 24.81 | 6.81 | - | 5.38 | 5.93 | 16.75 | 11.59 | 7.10 |
|  |  | Difference (%) | 15.3 | 12.7 | 34.3 | 6.8 | -0.3 | -0.1 | 80.2 | - | 8.1 | 15.0 | - | -31.7 | -28.2 | -22.7 | 17.3 | -24.4 |
|  | 5mm PTV expansion from GTV | No spacer | 42.88 | 36.71 | 28.03 | 89.64 | 57.94 | 27.94 | 0.77 | - | 25.15 | 7.75 | - | 7.83 | 8.19 | 19.74 | 10.90 | 12.18 |
|  |  | Spacer | 50.22 | 42.70 | 40.37 | 96.86 | 57.68 | 27.70 | 1.55 | - | 24.93 | 6.21 | - | 5.73 | 6.20 | 17.85 | 12.09 | 7.95 |
|  |  | Difference (%) | 17.1 | 16.3 | 44.0 | 8.1 | -0.4 | -0.9 | 101.3 | - | -0.9 | -19.9 | - | -26.8 | -24.3 | -9.6 | 10.9 | -34.7 |
| 3 mm large bowel PRV | 0mm PTV expansion from GTV | No spacer | 31.91 | 40.13 | 31.91 | 91.61 | 58.18 | 21.44 | 0 | 28.05 | 21.09 | 1.85 | 25.19 | 6.54 | 6.67 | 20.41 | 8.95 | 9.98 |
|  |  | Spacer | 41.77 | 44.71 | 41.77 | 98.86 | 57.16 | 25.08 | 0.16 | 28.02 | 21.26 | 1.43 | 24.45 | 4.95 | 6.06 | 16.85 | 9.90 | 7.70 |
|  |  | Difference (%) | 30.9 | 11.4 | 30.9 | 7.9 | -1.8 | 17.0 | - | -0.1 | 0.8 | -22.7 | -2.9 | -24.3 | -9.1 | -17.4 | 10.6 | -22.8 |
|  | 3mm PTV expansion from GTV | No spacer | 31.31 | 32.49 | 23.95 | 84.80 | 58.75 | 22.06 | 0 | 27.98 | 21.14 | 4.09 | 24.96 | 7.71 | 8.45 | 22.5 | 10.71 | 9.31 |
|  |  | Spacer | 47.47 | 44.55 | 40.01 | 98.05 | 58.06 | 24.96 | 0.25 | 28.18 | 21.40 | 2.85 | 25.00 | 5.03 | 5.55 | 17.76 | 12.10 | 7.88 |
|  |  | Difference (%) | 51.6 | 37.1 | 67.1 | 15.6 | -1.2 | 13.1 | - | 0.7 | 1.2 | -30.3 | 0.2 | -34.8 | -34.3 | -21.1 | 13.0 | -15.4 |
|  | 5mm PTV expansion from GTV | No spacer | 31.11 | 28.54 | 22.93 | 78.63 | 58.01 | 23.72 | 0.02 | 28.16 | 21.25 | 4.54 | 25.14 | 6.24 | 6.69 | 18.62 | 9.68 | 10.23 |
|  |  | Spacer | 48.25 | 40.73 | 37.09 | 90.43 | 58.10 | 24.56 | 0.13 | 28.06 | 21.40 | 3.23 | 24.93 | 5.26 | 5.91 | 18.42 | 12.87 | 8.46 |
|  |  | Difference (%) | 55.1 | 42.7 | 61.8 | 15.0 | 0.2 | 3.5 | 550.0 | -0.4 | 0.7 | -28.9 | -0.8 | -15.7 | -11.7 | -1.1 | 33.0 | -17.3 |
| 5 mm large bowel PRV | 0mm PTV expansion from GTV | No spacer | 31.53 | 39.28 | 31.53 | 91.46 | 58.33 | 21.63 | 0 | 27.92 | 19.28 | 0.62 | 25.18 | 6.7 | 6.9 | 22.39 | 9.73 | 11.19 |
|  |  | Spacer | 41.64 | 44.48 | 41.64 | 98.72 | 56.93 | 23.13 | 0 | 27.26 | 18.75 | 0.24 | 25.19 | 4.31 | 5.00 | 19.15 | 9.96 | 7.25 |
|  |  | Difference (%) | 32.1 | 13.2 | 32.1 | 7.9 | -2.4 | 6.9 | - | -2.4 | -2.7 | -61.3 | 0.0 | -35.7 | -27.5 | -14.5 | 2.4 | -35.2 |
|  | 3mm PTV expansion from GTV | No spacer | 26.4 | 27.61 | 22.50 | 79.19 | 58.96 | 22.34 | 0 | 28.195 | 18.91 | 0.49 | 25.11 | 8.13 | 8.67 | 23.01 | 10.42 | 10.41 |
|  |  | Spacer | 45.98 | 43.43 | 36.78 | 96.50 | 58.42 | 23.86 | 0.02 | 27.98 | 18.72 | 0.59 | 25.19 | 4.02 | 5.03 | 18.54 | 12.42 | 8.29 |
|  |  | Difference (%) | 74.2 | 57.3 | 63.5 | 21.9 | -0.9 | 6.8 | - | -0.8 | -1.0 | 20.4 | 0.3 | -50.6 | -42.0 | -19.4 | 19.2 | -20.4 |
|  | 5mm PTV expansion from GTV | No spacer | 26.49 | 24.80 | 21.83 | 72.00 | 58.48 | 23.4 | 0 | 27.91 | 19.21 | 0.55 | 24.87 | 7.08 | 7.59 | 19.32 | 10.32 | 9.95 |
|  |  | Spacer | 45.28 | 39.38 | 31.68 | 91.40 | 58.54 | 23.85 | 0.02 | 27.88 | 18.49 | 0.18 | 25.10 | 4.53 | 4.81 | 18.45 | 12.23 | 8.31 |
|  |  | Difference (%) | 70.9 | 58.8 | 45.1 | 26.9 | 0.1 | 1.9 | - | -0.1 | -3.7 | -67.3 | 0.9 | -36.0 | -36.6 | -4.5 | 18.5 | -16.5 |

Abbreviations: GTV, gross tumor volume; ITV, internal target volume; PRV, planning target volume at risk; PTV, planning target volume; SBRT, stereotactic body radiation therapy.

^*^Thecal sac PRV is thecal sac + 2mm, duodenum PRV is duodenum + 5mm
