# Supplementary material for: Utilization of a stabilized hyaluronic acid spacer in SBRT for retroperitoneal cancers: A case series and dosimetric analysis
Source: Clin Transl Radiat Oncol. 2025 Mar 8;52:100943. doi: 10.1016/j.ctro.2025.100943 (PMC11950742; doi:10.1016/j.ctro.2025.100943)
Supplement: Supplementary Data 9 [file mmc9.docx]

**Table S4.** Comparative dosimetric analysis for the right adrenal oligometastatic lesion using different PTV and large bowel PRV margins.

| Plans with different PTV and PRV margins with and without spacer | | | Targets | | | | | Large bowel | | Large bowel PRV | Small bowel | | ^*^Spinal cord | Spinal cord PRV | Duodenum | | | ^*^Duodenum PRV | Right chest wall | | Inferior vena cava | Left kidney | | | Liver | Liver-GTV | Skin Rind | |
| --- | --- | --- | --- | --- | --- | --- | --- | --- | --- | --- | --- | --- | --- | --- | --- | --- | --- | --- | --- | --- | --- | --- | --- | --- | --- | --- | --- | --- |
|  |  |  | GTV/ITV D99% (Gy) | PTV D95% (Gy) | PTV D99% (Gy) | PTV V40Gy (%) | D0.035cc (Gy) | D0.035cc (Gy) | D20cc (Gy) | D0.035cc (Gy) | D0.035cc (Gy) | D5cc (Gy) | D0.035cc (Gy) | D0.035cc (Gy) | D0.035cc (Gy) | D5cc (Gy) | D10cc (Gy) | D0.035cc (Gy) | D0.035cc (Gy) | D70cc (Gy) | D0.035cc (Gy) | D0.035cc (Gy) | D10% (Gy) | Dmean (Gy) | V10Gy (%) | Dmean (Gy) | D0.035cc (Gy) | D10cc (Gy) |
| No large bowel PRV | 0mm PTV expansion from GTV | No spacer | 32.33 | 35.86 | 32.33 | 88.38 | 54.90 | 31.98 | 9.79 | - | 7.60 | 0.71 | 6.24 | 7.63 | 9.54 | 6.82 | 5.28 | 9.57 | 12.09 | 0.84 | 32.46 | 9.95 | 6.64 | 2.38 | 1.87 | 1.03 | 8 | 3.32 |
|  |  | Spacer | 32.03 | 40.46 | 32.03 | 95.44 | 54.36 | 31.91 | 15.65 | - | 12.01 | 9.35 | 13.6 | 14.04 | 12.95 | 9.47 | 8.25 | 14.56 | 26.81 | 1.62 | 22.06 | 6.82 | 4.98 | 2.42 | 10.77 | 4.43 | 9.54 | 6.45 |
|  |  | Difference (%) | -0.9 | 12.8 | -0.9 | 8.0 | -1.0 | -0.2 | 59.9 | - | 58.0 | 1216.9 | 117.9 | 84.0 | 35.7 | 38.9 | 56.3 | 52.1 | 121.8 | 92.9 | -32.0 | -31.5 | -25.0 | 1.7 | 475.9 | 330.1 | 19.3 | 94.3 |
|  | 3mm PTV expansion from GTV | No spacer | 31.56 | 28.82 | 25.59 | 71.48 | 54.99 | 31.99 | 14.88 | - | 10.81 | 2.82 | 10.31 | 12.38 | 8.62 | 6.54 | 5.6 | 11.75 | 18.17 | 1.09 | 32.64 | 11.37 | 7.98 | 3.44 | 2.86 | 1.58 | 6.99 | 3.89 |
|  |  | Spacer | 32.1 | 31.9 | 27.25 | 79.96 | 54.81 | 31.82 | 17.78 | - | 14.33 | 10.04 | 14.57 | 15.42 | 15.1 | 11.1 | 9.75 | 17.08 | 29.77 | 3.13 | 25.43 | 9.28 | 7.4 | 3.98 | 15.46 | 5.69 | 10.14 | 6.96 |
|  |  | Difference (%) | 1.7 | 10.7 | 6.5 | 11.9 | -0.3 | -0.5 | 19.5 | - | 32.6 | 256.0 | 41.3 | 24.6 | 75.2 | 69.7 | 74.1 | 45.4 | 63.8 | 187.2 | -22.1 | -18.4 | -7.3 | 15.7 | 440.6 | 260.1 | 45.1 | 78.9 |
|  | 5mm PTV expansion from GTV | No spacer | 31.52 | 28.22 | 25.33 | 62.15 | 54.88 | 31.92 | 18.71 | - | 9.76 | 5.93 | 11.05 | 12.74 | 9.83 | 7.52 | 6.49 | 11.56 | 19.28 | 1.33 | 33.51 | 10.07 | 7.32 | 3.45 | 3.95 | 2 | 6.79 | 4.17 |
|  |  | Spacer | 31.93 | 29.71 | 25.67 | 73.11 | 54.92 | 31.93 | 18.99 | - | 14.51 | 10.68 | 15.61 | 16.49 | 14.98 | 11.16 | 9.67 | 17.33 | 33.06 | 3.82 | 27.89 | 9.52 | 7.89 | 4.42 | 19.78 | 6.63 | 10.13 | 10.68 |
|  |  | Difference (%) | 1.3 | 5.3 | 1.3 | 17.6 | 0.1 | 0.0 | 1.5 | - | 48.7 | 80.1 | 41.3 | 29.4 | 52.4 | 48.4 | 49.0 | 49.9 | 71.5 | 187.2 | -16.8 | -5.5 | 7.8 | 28.1 | 400.8 | 231.5 | 49.2 | 156.1 |
| 3 mm large bowel PRV | 0mm PTV expansion from GTV | No spacer | 31.65 | 33.64 | 32.37 | 69.16 | 52.29 | 31.94 | 12.99 | 37.62 | 11 | 1.94 | 12.3 | 11.18 | 9.86 | 6.86 | 5.46 | 10.51 | 11.7 | 0.79 | 23.9 | 8.32 | 5.9 | 2.25 | 2.35 | 1.42 | 6.12 | 3.54 |
|  |  | Spacer | 31.81 | 36.2 | 31.81 | 89.55 | 54.96 | 31.75 | 15.19 | 37.76 | 11.34 | 8.17 | 11.57 | 12.44 | 13.38 | 9.36 | 7.41 | 15.11 | 26 | 1.85 | 21.98 | 7.47 | 6.14 | 3.13 | 11.8 | 4.79 | 10.64 | 6.49 |
|  |  | Difference (%) | 0.5 | 7.6 | -1.7 | 29.5 | 5.1 | -0.6 | 16.9 | 0.4 | 3.1 | 321.1 | -5.9 | 11.3 | 35.7 | 36.4 | 35.7 | 43.8 | 122.2 | 134.2 | -8.0 | -10.2 | 4.1 | 39.1 | 402.1 | 237.3 | 73.9 | 83.3 |
|  | 3mm PTV expansion from GTV | No spacer | 31.61 | 29.96 | 28.54 | 61.65 | 54.28 | 31.95 | 16.15 | 37.97 | 11.33 | 4.17 | 12 | 13.12 | 8.02 | 6.65 | 5.92 | 8.63 | 13.82 | 1.08 | 29.58 | 8.45 | 6.45 | 2.9 | 3.27 | 1.77 | 5.76 | 3.59 |
|  |  | Spacer | 32.17 | 31.9 | 28.88 | 78.02 | 54.56 | 32 | 18.25 | 37.59 | 14.22 | 10.54 | 14.33 | 15.14 | 13.5 | 9.86 | 8.2 | 15.85 | 30.62 | 3.25 | 25.47 | 8.57 | 6.25 | 3.58 | 17.76 | 5.96 | 10.35 | 6.87 |
|  |  | Difference (%) | 1.8 | 6.5 | 1.2 | 26.6 | 0.5 | 0.2 | 13.0 | -1.0 | 25.5 | 152.8 | 19.4 | 15.4 | 68.3 | 48.3 | 38.5 | 83.7 | 121.6 | 200.9 | -13.9 | 1.4 | -3.1 | 23.4 | 443.1 | 236.7 | 79.7 | 91.4 |
|  | 5mm PTV expansion from GTV | No spacer | 31.24 | 29 | 27.62 | 56.7 | 52.46 | 31.82 | 19.06 | 37.92 | 11.47 | 5.37 | 11.62 | 11.62 | 12.81 | 8.54 | 6.16 | 9.67 | 18.31 | 1.39 | 32.53 | 9.03 | 6.5 | 3.09 | 3.53 | 1.91 | 5.26 | 3.89 |
|  |  | Spacer | 31.86 | 30.02 | 27.43 | 72.65 | 54.72 | 31.79 | 19.66 | 37.91 | 14.26 | 10.69 | 16.11 | 16.96 | 15.26 | 10.7 | 9.08 | 18 | 32.75 | 4.22 | 28.77 | 9.37 | 7.26 | 4.3 | 20.9 | 6.74 | 9.86 | 7.6 |
|  |  | Difference (%) | 2.0 | 3.5 | -0.7 | 28.1 | 4.3 | -0.1 | 3.1 | 0.0 | 24.3 | 99.1 | 38.6 | 46.0 | 19.1 | 25.3 | 47.4 | 86.1 | 78.9 | 203.6 | -11.6 | 3.8 | 11.7 | 39.2 | 492.1 | 252.9 | 87.5 | 95.4 |
| 5 mm large bowel PRV | 0mm PTV expansion from GTV | No spacer | 31.58 | 33.06 | 32.13 | 50.43 | 52.29 | 31.83 | 12.95 | 37.89 | 10.42 | 1.38 | 11.81 | 12.8 | 7.04 | 5.58 | 4.28 | 7.77 | 15.62 | 0.77 | 22.65 | 7.46 | 5.39 | 2.03 | 2.09 | 1.35 | 5.33 | 3.21 |
|  |  | Spacer | 29.74 | 33.66 | 29.74 | 83.72 | 54.685 | 31.35 | 14.7 | 31.35 | 12.18 | 9.55 | 11.67 | 12.44 | 11.69 | 8.4 | 7.16 | 13.32 | 26.75 | 8.4 | 22.14 | 7.8 | 6.08 | 2.99 | 12.29 | 4.91 | 9.92 | 6.24 |
|  |  | Difference (%) | -5.8 | 1.8 | -7.4 | 66.0 | 4.6 | -1.5 | 13.5 | -17.3 | 16.9 | 592.0 | -1.2 | -2.8 | 66.1 | 50.5 | 67.3 | 71.4 | 71.3 | 990.9 | -2.3 | 4.6 | 12.8 | 47.3 | 488.0 | 263.7 | 86.1 | 94.4 |
|  | 3mm PTV expansion from GTV | No spacer | 31.53 | 30 | 28.83 | 45.23 | 50.83 | 31.72 | 17.05 | 37.9 | 13.04 | 3.68 | 13.77 | 15.11 | 6.37 | 5.27 | 4.55 | 8.04 | 17.84 | 1 | 28.01 | 9.11 | 6.82 | 3 | 3.34 | 1.76 | 5.96 | 3.84 |
|  |  | Spacer | 31.95 | 31.33 | 29.35 | 74.61 | 54.83 | 31.97 | 18.47 | 37.95 | 13.54 | 10.25 | 14.63 | 15.46 | 13.24 | 9.71 | 7.47 | 14.84 | 30.75 | 3.33 | 24.73 | 8.98 | 6.99 | 4.09 | 16.78 | 5.89 | 10.03 | 7 |
|  |  | Difference (%) | 1.3 | 4.4 | 1.8 | 65.0 | 7.9 | 0.8 | 8.3 | 0.1 | 3.8 | 178.5 | 6.2 | 2.3 | 107.8 | 84.3 | 64.2 | 84.6 | 72.4 | 233.0 | -11.7 | -1.4 | 2.5 | 36.3 | 402.4 | 234.7 | 68.3 | 82.3 |
|  | 5mm PTV expansion from GTV | No spacer | 31.33 | 29.21 | 27.69 | 45.46 | 52.42 | 31.51 | 18.77 | 37.81 | 11.66 | 6.17 | 13.79 | 14.96 | 7.91 | 5.93 | 5.26 | 8.74 | 18.48 | 1.28 | 31.04 | 9.58 | 7.07 | 3.34 | 3.86 | 1.96 | 6.46 | 4.02 |
|  |  | Spacer | 31.68 | 29.76 | 27.81 | 67.49 | 54.76 | 31.82 | 19.87 | 37.94 | 13.78 | 10.91 | 17.03 | 17.88 | 13.75 | 10.14 | 8.61 | 15.97 | 34.3 | 4.27 | 26.81 | 9.88 | 8.37 | 4.99 | 20.7 | 6.8 | 9.49 | 7.29 |
|  |  | Difference (%) | 1.1 | 1.9 | 0.4 | 48.5 | 4.5 | 1.0 | 5.9 | 0.3 | 18.2 | 76.8 | 23.5 | 19.5 | 73.8 | 71.0 | 63.7 | 82.7 | 85.6 | 233.6 | -13.6 | 3.1 | 18.4 | 49.4 | 436.3 | 246.9 | 46.9 | 81.3 |

Abbreviations: GTV, gross tumor volume; ITV, internal target volume; PRV, planning target volume at risk; PTV, planning target volume; SBRT, stereotactic body radiation therapy.

^*^Spinal cord PRV is spinal cord + 2mm, duodenum PRV is duodenum + 5mm
